# The Public Health Perils of Search Engine Marketing: Insights for Research and Regulation

**DOI:** 10.34172/ijhpm.9180

**Published:** 2025-09-14

**Authors:** Alessandro R. Marcon, Marco Zenone

**Affiliations:** ^1^Health Law Institute, Faculty of Law, University of Alberta, Edmonton, AB, Canada.; ^2^Faculty of Health Sciences, University of Ottawa, Ottawa, ON, Canada.

## Introduction

 As the world moves increasingly online, media and technology companies’ power to influence grows exponentially. So too does the power of companies that can effectively navigate and manipulate the structures governing internet activity. Learning how media elements like sponsored ads in Google search results can be manipulated for commercial gains and power of influence is valuable to understanding how health science information circulates online and what impacts result from such activity.

 More than 67% of the world’s population is now on the internet, and over 93% of those online are monthly social media users.^1^ From mental health^2^ to misinformation^3^ to commercialized health promotion,^4,5^ extensive research has examined the complex impact that increased online activity is having on diverse populations’ health and well-being. And yet for all the research insights garnered on human behaviour, the inner-workings of media companies’ algorithms remains opaque. This is because the structural algorithms of the most popular websites such as Google’s search bar, TikTok, and YouTube is the central element of their product’s experience and remains a proprietary formula. Beyond the dissemination of highly-entertaining videos, TikTok, for example, has become famous for its uncanny addictiveness – a characteristic often attributed to its algorithm’s precise ability to match content with audiences’ viewing desires.^6^ Google’s Page Rank and search relevance algorithms govern what occupies the first page, or first appearing results of the world’s most used search engine.^7^ This page-rank reality is also rapidly changing with artificial intelligence (AI) responses from Google’s Gemini now often appearing before search results, which in turn, arguably gives even greater weight to Google’s role in information selection. Recent decisions made by Google to enable indexing of social media content in search results require careful and ongoing monitoring, considering that commercialized content from high profile social media influencers could gain heightened prominence in users’ search results.^8^

 Given Google’s highly impactful role in selecting and disseminating information in response to users queries, there is no longer a serious marketing team that does not strive for search engine optimization that can increase traffic to company websites. The value of appearing near the top of Google’s search results is well known. Some might also be aware that sponsored search results appear alongside other media content returned from search queries. Few might be aware, however, that companies have purchasing power in appearing on the top of the sponsored results pile by making use of the Google Ads mechanism.^9^ In this viewpoint, we outline problematic aspects of this purchasing power, arguing for the public health importance of understanding its mechanism. In response, we outline a research agenda that can offer value to the design and implementation of research, public health policy, and government regulation.

## Advertising With Google’s Search Bar

 Online advertising is primarily concerned with gaining presence in coveted spaces, and increasing traffic to commercial websites where sales can occur. In 2026, a 30-second advertisement during the International Federation of Association Football World Cup soccer championship- one of the most viewed events in the world – is expected to cost approximately $US 7 to $US 8 million.^10^ Like a World Cup television ad, occupying space on Google’s first page of search results is a desired advertising objective. Unlike a World Cup ad, appearing on Google’s first page does not entail purchasing a time slot but rather paying for the phrases used in Google searches that generate results. That is, unlike a fixed space in a live streamed event—where, albeit some time slots are more desired than others—taking advantage of Google search results requires anticipating searched-for phrases and matching ads accordingly. Applying the World Cup analogy to Google searches would result, for example, in a potato chip company paying for their sponsored advertisements to appear when people search for phrases such as “best World Cup party snack,” or “best potato chip flavour.” A health supplement company, for example, might wish to pay for the spaces that accompany searches for the “importance of health supplements,” “vitamin supplements for older people,” “best health supplements,” or “supplements for leaky gut.” Another wellness company might target searches such as “supplements for cancer therapies,” “beautiful skin,” “protein for muscle growth,” or “training recovery.” Of course, it is not the case that companies purchase these phrases on the Google search bar, becoming proprietary owners of their use. Rather, companies bid to gain as high a ranking as possible on Google’s search result list.^9^

## Harmful Marketing Practices

 A marketing approach that aligns products and services with search phrases might appear to be a standard, assumed, even innocuous practice. If people are searching for potential products and services, it is expected that companies would compete for the spaces that provide responses to such queries. There are numerous cases, however, where this practice becomes exploitative, raising a host of public health issues. Detailed below are some relevant examples and contexts that we have researched and observed in our research/professional activities. These cases should not be interpreted as an exhaustive list, or even the most representative, of how Google Ads could function determinately. Indeed, seemingly any industry actor could use the mechanism with malicious effect, and it is the objective of this manuscript to encourage research that examines how Google Ads are used in a range of health contexts.

###  Scientifically Unsupported and Harmful Medical Treatments

 Businesses offering unproven and harmful treatments for serious or life-threatening illnesses like cancer may purchase—as was observed in our research—the advertising space with sensitive Google searches such as “stage 4 cancer treatment,” “best cancer therapy,” or “effective alternatives to chemotherapy.”^11^ Of concern, Google users searching for treatment options for an unmet need for any illness, ranging from multiple sclerosis, to autism, can be shown links to harmful entities providing scientifically unsupported treatment.

###  Public Health Information Provided by Organizations With a Vested Interest

 Individuals seeking solutions for gambling, drug, or alcohol addictions could be shown not only misleading approaches but promotional material for the very activity they are striving to avoid. This does not mean ads for specific products, but rather, information provided by industry-funded organizations undermining evidence-based and regulatory interventions. For example, Drinkaware, an industry funded charity in the United Kingdom, provides questionable education-focused alcohol addiction information to individuals seeking support.^12^ In other cases, astroturf groups, or lobbying organizations, may undermine consensus scientific positions, such as denying climate change. Google has hosted ads which use search phrases like “climate change is a hoax” from alternative media outlets.^13^

###  Sensitive Personal Decisions

 Persons seeking information or advice on personal decisions, such as abortion, may be met with misleading messaging. For example, anti-abortion centers monetized Google search phrases seeking to access abortion services.^14^ In other cases, people searching for weight loss solutions might be shown ineffective solutions or potentially, in the case of some drugs, off-label effects which may be harmful.^15^ Similarly, baby formula companies, whose marketing practices have highlighted as problematic by research communities,^16^ could make their products present in search results when new parents, whose child may be experiencing colic, are searching for information on breastfeeding support. Formula companies, as well, may obscure the benefits of breastfeeding to parents deciding whether to breastfeed or use formula.

 These cases present some harmful scenarios, and more research is required across a range of global health contexts to accurately map out the extent of issues and where intervention might be most pressing. These described cases, however, demonstrate potential for real public health harm with serious and potentially legal implications. First, responses to vital and sensitive health queries can be tainted by misinformation. Accurate, evidence-based information in trustworthy sources can lose relevance when presented alongside financial solutions presented in an easy-to-fix manner. Public health can suffer as a result, and individuals might suffer financial exploitation. Second, advertising unproven, ineffective, or harmful products and services can present breaches of regulatory and legal frameworks. Third, in addition to the potential of public health damage, individuals may be directed towards ideological or politically charged sources that promote inaccurate scientific information alongside conspiracy theories and unfounded misinformation that generates anxieties and mistrust of public health institutions.

###  Opportunities and Challenges in Mapping out Google Search Advertising 

 Paying for Google Search presence via bidding on phrases is not inherently a practice that harms public health. It is a tool used by industry to advertise, and can even, in some cases, work towards helping consumers find desired and effective products for their needs, for example exercise or sporting equipment. The issues, exist, however in how that tool is used and how transparent the specifics of those activities are. Currently, it is far from a transparent activity. In fact, it can be difficult to know when and how it is taking place. There are tools, however, that can assist. Software like Semrush, for example, can provide details around which companies are bidding on which search phrases. It is possible to see not only which search phrases were selected but how much financial expenditure was attributed to each. Analysis of these search phrases can illustrate potential conflicts with regulatory, legal, or ethical frameworks. With such software, however, coverage capabilities might be lacking as it is not always possible to see the bidding activity of all companies. As such, increased access to these marketing activities can be a goal of regulatory efforts. Surveillance activities can lead the way to determining the cases and contexts which require the applications of current regulatory measures, or potentially the creation of new ones, to curb their harmful relevance.

 Powerful entities such as Google play an increasingly influential role in information dissemination. In response, regulatory efforts have been undertaken across the world to manage these powerful roles that media and technology companies occupy. Australia’s “Media Bargain Code,” and Canada’s proposed bill C-18, for example, strive to increase wealth distribution among news organizations and journalists.^17,18^ As public health organizations work towards accurate information dissemination among diverse publics, increasing transparency of major platforms’ inner workings remains vital. Understanding broadly, and in specific cases, how industry can take advantage of Google search queries stands as a core component of these public health efforts. The next step, of course, is designing and implementing tools to address it. Relatively straightforward measures could include restricting the ads mechanism from being used in particular contexts; that is, restricting sponsored ads to appear alongside queries associated with vulnerable populations or in serious, high-risk contexts such as life-threatening diseases. Certain keywords and phrases could be prohibited from use in targeted ads, and AI tools could be deployed to help regulatory bodies with surveillance activities. Specific companies with a history of egregious marketing practices could be banned from using the mechanism altogether. Lastly, search engine algorithms could be designed to give prominence only to websites supported by accurate scientific evidence, in particular non-profit public organizations with the objective of pursing public health goals and not profits. Core to having media companies change the effect of algorithms is public entities and research communities clearly articulating the (severity of) public health harms related to how algorithms currently function. As a result, harder regulatory approaches from governing bodies could include the use of bans or financial penalties.

 In summary, top ranking google search results contain sought after advertising spaces. The Google Ads mechanism enables industry to bid on users’ search queries, whereby highest bidders gain access to advertise products and services appearing with top search results. Targeting search queries for commercial advertising raises numerous public health issues such as presenting scientifically unsupported and harmful medical treatments, highlighting information from parties with invested interests, and misleading the public seeking solutions to sensitive personal health issues. Harmful impacts from search engine marketing should be understood and addressed by researchers, public health agencies, and relevant regulatory bodies. Tools exist to conduct these analyses and should be utilized by researchers the world over in relation to pressing health contexts for particular regions and demographics.

## Acknowledgements

 We wish to acknowledge the support of our research team in particular Timothy Caulfield and Robyn Hyde-Lay and its network of collaborators.

## Ethical issues

 Not applicable.

## Conflicts of interest

 Authors declare that they have no conflicts of interest.
